# Triple-negative breast cancer at Helen Joseph Hospital: Prevalence, age and imaging features

**DOI:** 10.4102/sajr.v29i1.3247

**Published:** 2025-10-16

**Authors:** Tsholofelo Zondi, Grace Rubin, Carol-Ann Benn, Sharadini K. Gounden

**Affiliations:** 1 Department of Radiology, Faculty of Health Sciences, University of the Witwatersrand, Johannesburg, South Africa; 2 Department of Immunology, Faculty of Health Sciences, University of Pretoria, Pretoria, South Africa; 3 Discipline of Radiology, School of Clinical Medicine, College of Health Sciences, University of KwaZulu-Natal, Durban, South Africa

**Keywords:** triple-negative breast cancer, breast neoplasms, imaging characteristics, Ki-67 antigen, early-onset breast cancer, epidemiology, mammography, ultrasound

## Abstract

**Background:**

Triple-negative breast cancer (TNBC) is considered an aggressive subtype, defined by the absence of oestrogen, progesterone and HER2 receptors. It typically presents earlier and more aggressively. Limited data exist on its prevalence, age of onset and imaging features in South Africa.

**Objectives:**

This study aimed to assess the prevalence of TNBC at Helen Joseph Tertiary Hospital (HJTH), describe its histopathological features and explore trends in age at diagnosis and imaging patterns—including early-onset disease.

**Method:**

A retrospective review of 280 female patients with histologically confirmed breast cancer, diagnosed between January 2021 and December 2023, was conducted. Demographic, imaging and histopathology data were analysed using descriptive statistics and chi-square tests.

**Results:**

The diagnosis of TNBC accounted for 17% (48/280) of all breast cancer cases in the cohort. The TNBC lesions typically measured 1–5 cm and showed nodal involvement in 73% of cases. Despite their aggressive biology, many TNBC lesions appeared circumscribed or only mildly irregular on imaging, mimicking benign masses. Among all the 280 breast cancer cases, 61% were high-grade. The mean Ki-67 index for TNBC was the highest at 52%, followed by HER2+ (39%), Luminal B (33%) and Luminal A (21%). Notably, some HER2+ and TNBC cases exhibited lower Ki-67 indices, highlighting heterogeneity within these subtypes.

**Conclusion:**

This study highlights the complexity of breast cancer presentation in a South African setting, particularly the discordance between tumour biology and imaging.

**Contribution:**

These findings contribute local data on TNBC in an urban public healthcare context, supporting improved imaging awareness and clinical vigilance in resource-limited settings.

## Introduction

Breast cancer remains a prevalent cancer among women worldwide and is a leading cause of cancer-related mortality.^1^ Incidence rates vary by region, with higher rates observed in high-income countries (HICs).^2^ Notably, age-standardised mortality from breast cancer in HICs declined by 40% between the 1980s and 2020s, largely because of improvements in early detection and treatment.^3^ In contrast, low- and middle-income countries (LMICs) have not seen comparable declines in mortality. Many have experienced increases, driven by several factors: late-stage diagnosis, which limits effective treatment and often results in poorer treatment responses; limited access to tailored therapies; a lack of organised screening programmes; and weaknesses in healthcare infrastructure.^4,5^

Historically, breast cancer in LMICs has been characterised as more aggressive, with studies reporting higher rates of triple-negative disease and lower hormone receptor positivity.^6,7^ However, more recent analyses suggested that these patterns may be partially attributed to variability in receptor testing quality.^8^ Other indicators of tumour aggressiveness include high histological grade, elevated Ki-67, HER2 positivity and early age at diagnosis. Understanding the local distribution of such tumour characteristics in resource-limited settings is critical for improving outcomes and guiding public health strategies.^9,10,11^

Breast cancer is a heterogeneous disease composed of distinct molecular subtypes that influence prognosis and guide treatment decisions.^11^ Luminal A tumours are oestrogen receptor (ER)-positive with low proliferation indices (Ki-67 < 20%), typically low grade and respond well to endocrine therapy.^12^ Luminal B tumours are also ER-positive, but demonstrate higher proliferation and may express HER2, often requiring additional systemic treatment.^12^ The HER2-enriched tumours, defined by HER2 overexpression, tend to be more aggressive but are amenable to targeted HER2 therapies.^13^ Triple-negative breast cancer (TNBC), which lacks ER, PR and HER2 expression, is associated with a more aggressive clinical course, frequently affects younger patients and lacks targeted therapy options, although some immunotherapy options show promise.^14^ Despite this, TNBC can exhibit benign imaging characteristics that complicate early detection.^15^ Accurate molecular classifications are vital, especially in resource-limited settings.

Early-onset breast cancer—diagnosed in women under 40—has been linked to aggressive biological features such as high-grade histology and elevated proliferation markers. While TNBC is often associated with this age group in high-income settings,^16^ emerging evidence suggests that poorer outcomes in younger patients may be driven more by delayed diagnosis than by tumour biology itself.^17^ Notably, a recent local audit found that Luminal A and B subtypes were most common among younger South African women—a distribution that contrasts with international trends where TNBC tends to predominate. This divergence further challenges the assumed link between early-onset breast cancer and TNBC in all populations.^18^

Despite growing international research, South African data on TNBC remain limited, particularly regarding radiological features. As radiology is often the first point of contact in breast cancer diagnosis, characterising the imaging appearance of TNBC is vital. This study aims to assess the prevalence, imaging characteristics and histological profile of TNBC at a public hospital serving an urban South African population.

## Research methods and design

This retrospective, cross-sectional study included female patients with histologically confirmed breast cancer diagnosed between 01 January 2021 and 31 December 2023. Patients were seen or referred to Helen Joseph Tertiary Hospital (HJTH) Breast Unit in Johannesburg, South Africa. Only patients with complete receptor status and accessible imaging reports were included. Male patients and those with missing data were excluded from the sample.

Patients were identified *via* hospital records and the Picture Archiving and Communication System (PACS). Demographic data (age at diagnosis), tumour characteristics (histological subtype, grade, Ki-67 index, receptor status) and imaging findings (from ultrasound and mammography reports) were extracted. Where available, details on lesion size, shape, margins, posterior features and lymph node involvement were recorded.

Histopathology results, including receptor profiling and Ki-67 index, were obtained from the National Health Laboratory Service (NHLS) online system. Breast cancer subtypes were categorised into Luminal A, Luminal B, HER2-enriched and TNBC based on standard immunohistochemistry groupings.

Liver ultrasound findings and other staging data were reviewed when included in the reports. As per institutional protocol, liver imaging is routinely performed for suspected or confirmed breast cancer. However, not all reports provided complete staging information.

### Data analysis and statistics

Data were captured anonymously using a structured data collection sheet. Repeat entries were identified and removed before analysis. Data were analysed using Microsoft Excel and Stata. Descriptive statistics were used to calculate frequencies, means and proportions. Chi-square tests were employed to assess associations between molecular subtype and tumour grade. Numerical variables such as Ki-67 index and age at diagnosis were summarised using means and standard deviations (s.d.).

### Ethical considerations

Ethical approval was obtained on 20 March 2024 from the University of Witwatersrand Human Research Ethics Committee (HREC). Study approval number: M2400348. Patient data were anonymised and stored in a password-protected computer and database to maintain confidentiality.

## Results

**FIGURE 1:**
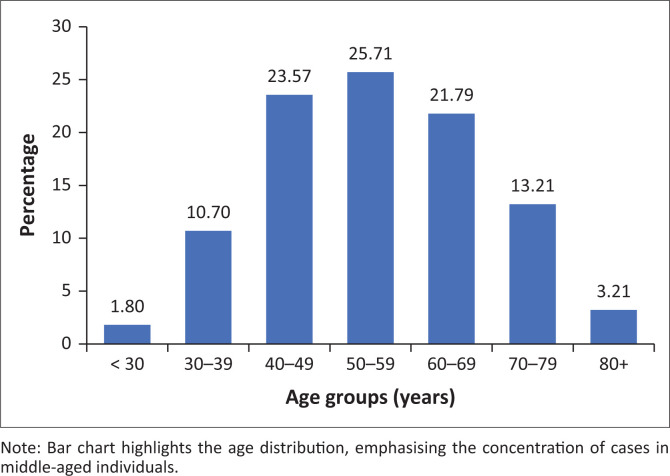
Age distribution of patients.

The study included 280 patients aged 27 to 95 years (median ± s.d., 54 ± 13.6). Most patients were in the middle-age range, with very few under the age of 30 years (Figure 1).

### Molecular subtype distribution and histopathological features

**FIGURE 2:**
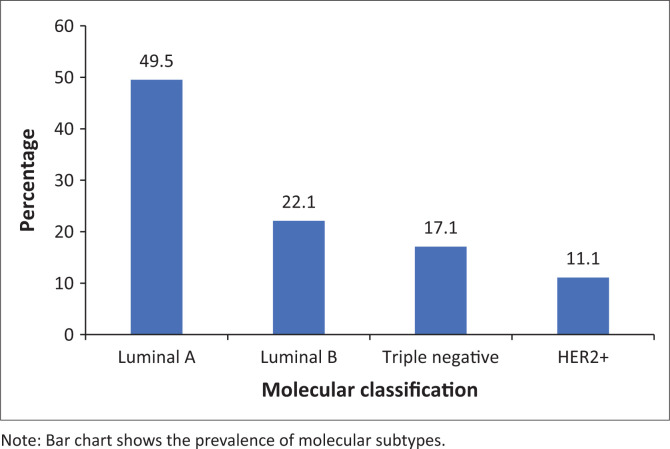
Molecular classification of breast cancers.

Luminal A and Luminal B were the most common molecular subtypes. Triple-negative breast cancer and HER2+ were less frequent (Figure 2). Triple-negative breast cancer exhibited the highest tumour grade and proliferation index (92% and 52%, respectively), followed by HER2+ and Luminal B. Luminal A tumours were typically of low grade with lower Ki-67 levels (Table 1). The association between molecular subtype and tumour grade was statistically significant (*p* < 0.001).

**TABLE 1:** Histopathological features across molecular subtypes.

Molecular classification	Mean Ki-67 (%)	High-grade tumours (Grade 3)	Total cases	High-grade tumours (%)
HER2+	38.8	19	31	61.3
Luminal A	21.5	30	138	21.7
Luminal B	33.4	25	62	40.3
Triple negative	52.0	44	48	91.7

### Triple-negative breast cancer findings

**FIGURE 3:**
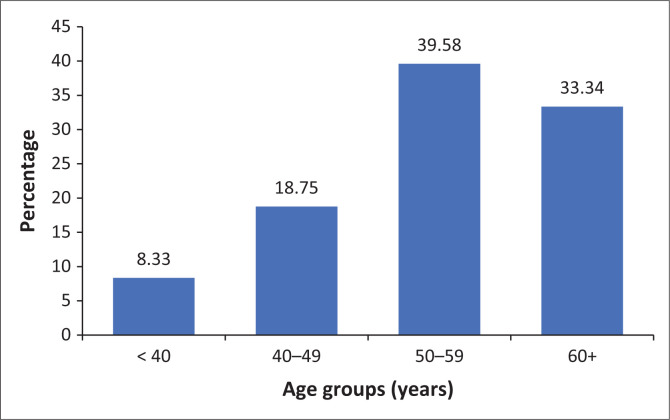
Age distribution of triple-negative breast cancer cases.

Triple-negative breast cancer was most frequently diagnosed in patients in the 50–59-year age group. There was no statistically significant association between age group and TNBC diagnosis (*χ*^2^ = 9.75, *p* = 0.083, df = 5) (Figure 3).

**FIGURE 4:**
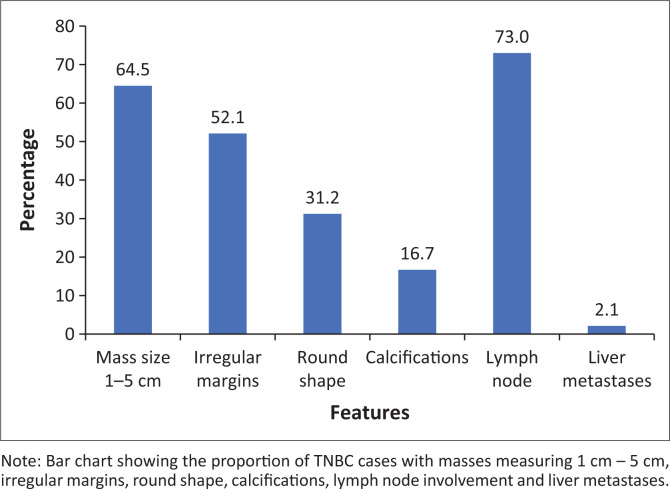
Imaging and clinical features of triple-negative breast cancer (TNBC).

**FIGURE 5:**
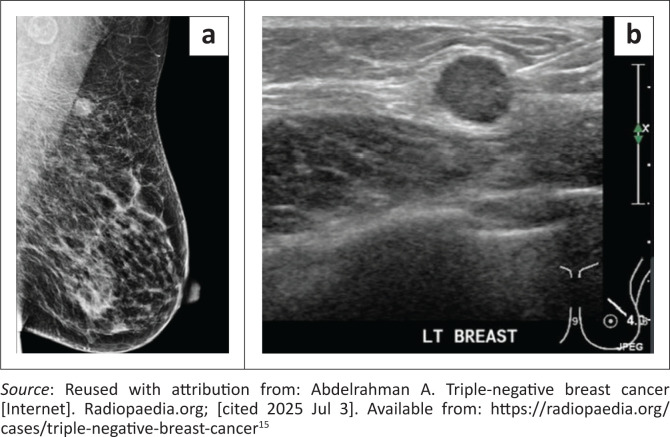
(a) Mediolateral oblique (MLO) mammographic view of the left breast of a 50-year-old female, demonstrating a round, circumscribed intermediate-density mass in the upper outer quadrant with an ipsilateral axillary lymph node. The lesion mimics benign morphology, although such roundness can be more common in triple-negative breast cancer (TNBC) than fibroadenoma.^15^ (b) Ultrasound image of the left breast in the same patient showing a round, circumscribed, hypoechoic mass with posterior acoustic enhancement. While this morphology resembles a benign fibroadenoma, the round shape and the presence of subtle microlobulated margins should raise suspicion for malignancy—features more commonly seen in TNBCs than in true fibroadenomas.^15^

Triple-negative breast cancer lesions most often presented as solid, irregular masses measuring 1–5 cm. While irregular shapes predominated, a notable number of lesions were round, with fewer appearing oval. Calcifications were uncommon. Lymph node involvement was frequent, while liver metastases were rare (Figure 4). On mammography, lesions commonly appeared as indistinct, irregular masses without architectural distortion (Figure 5a). On ultrasound, they were hypoechoic with indistinct margins and posterior acoustic enhancement (Figure 5b).

### Early-onset breast cancer

**FIGURE 6:**
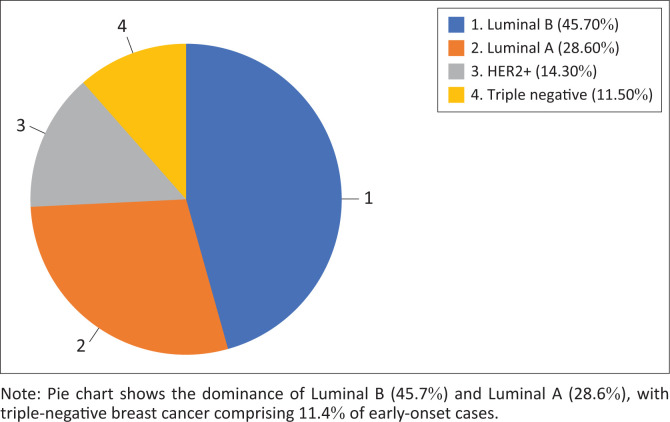
Early-onset breast cancer by molecular subtype.

Patients under 40 years made up a small proportion of the cohort (12%). Among these, Luminal B was the most common subtype (46%), followed by Luminal A (29%). Triple-negative breast cancer accounted for 11% of early-onset cases (Figure 6).

**FIGURE 7:**
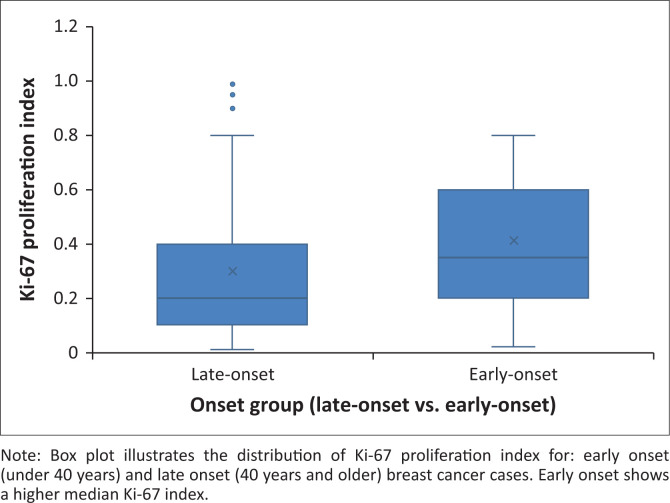
Ki-67 proliferation index by age category.

High-grade tumours were seen in 47% of the early-onset cases, compared to 41% in the late-onset patients. The Ki-67 proliferation index was significantly higher in early-onset breast cancer (median: 40%) than in late-onset cases (median: 25%), with this difference reaching statistical significance (*p* = 0.0036) (Figure 7).

## Discussion

This study provides insight into the imaging and histopathological profile of breast cancer in a South African tertiary care setting, with a specific focus on TNBC and early-onset disease. While the molecular subtype distribution is broadly consistent with international data, the cohort shows a high prevalence of aggressive tumour features—particularly elevated Ki-67 indices and histological grade—across multiple subtypes. These results validate global findings on TNBC biology and expand their relevance to a South African population, where resource constraints and diagnostic variability may compound clinical outcomes. The study’s integration of imaging and histopathological data further enhances its relevance for diagnostic optimisation in similar contexts. Triple-negative breast cancer comprised 17% of cases, aligning with global prevalence estimates of 10%–20%.^12,13^ These tumours demonstrated hallmark features of biological aggression—92% were Grade 3 and the mean Ki-67 index was 52%, which is consistent with the global literature describing TNBC as a poorly differentiated, highly proliferative entity.^14^

Interestingly, the highest proportion of cases occured in the 50–59 years age group, with relatively fewer cases diagnosed in women over 60 years. This contrasts with international trends, where incidence typically peaks in the 60s. The discrepancy may reflect differences in health-seeking behaviour, limited access to screening mammography or underdiagnosis in older women in this setting.

Although not overrepresented, TNBC’s imaging and histological profiles reflect notable diagnostic challenges. Most TNBC lesions appear as moderate-sized, irregular masses on imaging, with lymph node involvement in 73% of cases. However, the imaging features frequently mimicked benign lesions: many appeared circumscribed or only mildly irregular, demonstrating posterior acoustic enhancement without significant spiculation or distortion. This is likely because of the tumour’s rapid growth, which outpaces the development of a desmoplastic stromal response—a key feature often responsible for the classic radiological hallmarks of malignancy.^15^ These findings highlight a potential diagnostic pitfall: TNBC can resemble fibroadenomas, particularly in younger women, delaying definitive diagnosis, unless clinical and histological correlation is pursued. There are, however, subtle imaging clues that may raise suspicion: lesion roundness as well as microlobulated margins should not be dismissed. When present, these features may support earlier biopsy in cases initially presumed benign.

Only 2% of TNBC cases demonstrated liver metastases, which contrasts with global trends where visceral involvement is more frequent in advanced disease.^11^ This may reflect early-stage detection, underreporting or the limited sensitivity of ultrasound-based staging.^18^ This observation warrants further exploration to determine whether it reflects true biological behaviour, limitations in staging sensitivity or contextual factors unique to this setting. There was no statistical association between TNBC and age distribution (*p* = 0.083), however, the sample size likely limited statistical power.

Early-onset disease represented 12% of the cohort, which is in keeping with global trends.^17,18^ Contrary to common assumptions linking younger age with TNBC, Luminal B (46%) and Luminal A (29%) were the most prevalent subtypes in this group, with TNBC comprising only 11%. These findings are consistent with those of Chaane et al., suggesting that subtype distribution in younger patients may be influenced by population-specific factors such as genetics or referral patterns.^14,18,19^

Although TNBC was not dominant among early-onset cases, this group still exhibited aggressive biological features: 49% were high-grade tumours, and many had high Ki-67 indices. These patterns highlight the importance of early detection and age-tailored treatment pathways, particularly in low-resource settings where younger patients may present later because of reduced screening.

### Study’s limitations

The modest cohort size limited statistical robustness, particularly for subgroup analysis of TNBC. Additionally, as the study cohort consisted of symptomatic patients referred for imaging and biopsy, rather than a randomly selected population, selection bias may have influenced observed age and subtype distributions.

Data about liver metastases were lacking on many reports, while reliance on ultrasound may also have led to underreporting of distant metastases in many cases. These factors may have decreased the accuracy of the results, particularly pertaining to staging at diagnosis.

The absence of a second radiological reader and reliance on available records limited standardisation.

### Future applications

Many TNBC lesions mimicked benign findings, increasing the risk of delayed diagnosis—especially in younger women. At the study hospital, presumed fibroadenomas are usually monitored with imaging every 6 months for up to 18 months; biopsy is only indicated if the lesion enlarges. Yet in the study’s cohort, several TNBC cases appeared round and vertically oriented—features more typical of malignancy than of true fibroadenomas. Adding red flags like round shape, vertical orientation and subtle microlobulations to structured reporting templates could prompt earlier biopsy. This may help radiologists to distinguish TNBC from benign lesions and reduce diagnostic delays. A prospective study could assess how well these features improve early detection in resource-limited settings.

The unexpectedly low rate of distant metastases, especially to the liver, may reflect underdiagnosis because of incomplete staging or inconsistent documentation rather than true disease behaviour. A prospective study incorporating routine CT or MRI staging at diagnosis could clarify this trend and help refine staging protocols in similar settings.

Finally, the high proportion of Luminal B tumours among younger women reinforces the need for tailored care pathways that integrate endocrine therapy, fertility preservation and psychosocial support. This population has distinct clinical and reproductive considerations that merit specific treatment planning.

## Conclusion

This study highlights the complexity of breast cancer presentation in a South African setting, particularly the interplay between clinical, radiological and pathological findings, and how these may contribute to diagnostic uncertainty. While TNBC prevalence was in line with global norms, high-grade features and elevated Ki-67 indices were observed across subtypes. The imaging–pathology mismatch in TNBC is particularly relevant and may contribute to diagnostic delay. Together, these findings reinforce the need for early detection, more sensitive imaging tools, a higher clinical and radiological index of suspicion and greater awareness of population-specific tumour patterns in resource-limited settings.
